# Celiac disease in patients with type-1 diabetes mellitus screened by tissue transglutaminase antibodies in northwest of Iran

**DOI:** 10.4103/0973-3930.44081

**Published:** 2008

**Authors:** Nasrin Sharifi, Manouchehr Khoshbaten, Akbar Aliasgarzade, Amir Bahrami

**Affiliations:** 1 Department of Nutrition, Faculty of Health and Nutrition, Tabriz University of Medical Sciences, Golgasht Street, Tabriz, Iran; 2 Department of Gastroenterology and Hepatology, Drug Applied Research Center, Tabriz University of Medical Sciences, Iran; 3 Department of Endocrinology, Emam Reza University Hospital, Tabriz University of Medical Sciences, Iran

**Keywords:** Celiac disease, tissue transglutaminase antibodies, type 1 diabetes

## Abstract

**BACKGROUND:**

High prevalence rates of celiac disease (CD) in patients with type-1 diabetes mellitus (T1DM) have been reported in several countries. However, the data regarding this association are scarce in Iran. In this study, we report the prevalence of CD in patients with T1DM in northwest of Iran using tissue transglutaminase antibodies (tTGA) as a screening test.

**METHODOLOGY:**

One hundred patients with T1DM (58 women and 42 men) aged 21.8 ± 8.86 years (age range: 7–50 years) were compared with 150 healthy people (82 women and 68 men) aged 28.9 ± 9.07 years (age range: 4–50 years). All subjects were serologically screened for the presence of tTGA. Total immunoglobin A (IgA) was obtained to investigate IgA deficiency. Subjects positive for tTGA and deficient for IgA were submitted to upper gastrointestinal endoscopy.

**RESULTS:**

Eight patients with T1DM (8%) and three of the controls (2%) were positive for tTGA (*P* = 0.023), while only 3% of the tTGA positive T1DM patients underwent duodenal biopsy and all of them showed partial or total villous atrophy. The mean age of tTGA positive cases was significantly lower than tTGA negative ones (mean difference 7.17; 95% CI: 0.82–13.52). None of the tTGA positive T1DM patients had a history of chronic diarrhea, but one out of eight tTGA positives reported history of dermatitis (*P* = 0.001). Also, none of the tTGA positive subjects presented IgA deficiency. There was a significant difference in history of chronic diarrhea (*P* = 0.006) and autoimmune diseases (*P* = 0.001) between patients with T1DM and controls.

**CONCLUSION:**

This study showed higher prevalence of CD in patients with T1DM than in general population of northwest Iran and the data lend support to recommend regular screening for CD in all patients with T1DM.

## Introduction

Type-1 diabetes mellitus (T1DM) is a chronic autoimmune disorder with varying degrees of insulin deficiency resulting from an immune-mediated destruction of pancreatic β-cells, usually presenting in young individuals.[1] T1DM can be associated with other clinical, subclinical, or potential organ-specific autoimmune diseases. Celiac disease (CD) is an autoimmune enteropathy induced by gluten proteins present in wheat, barley, rye; and characterized by small intestinal lesions of variable severity.[2] In its classic form, CD appears with symptoms and signs of intestinal malabsorption. However, the disease may occur in a silent or latent form.[3] Co-existence of T1DM and CD was first suspected in 1954.[4] The same ‘susceptibility genotypes’ are involved in the etiopathogenesis of diabetes mellitus and CD. In both diseases, genetic susceptibility is associated with the HLA-DQ α1*0501, β1*0201 heterodimer, which preferentially presents gluten-derived gliadin peptides on its antigen-presenting groove to stimulate intestinal mucosal T cells.[5] With the existing identical gene location in both diseases, it seems that CD is more frequent in patients with T1DM than in general population. Using different screening procedures for auto antibodies, the reported prevalence of CD in patients with T1DM ranged from 0.6–16.4%.[6] Among different types of serological tests for screening CD, such as anti-gliadin antibodies (AGAs) and antiendomysial IgA antibody (EMA), tissue transglutaminase antibodies (tTGA) has proved to be a very specific indicator to identify subjects with latent CD.[7] It is well known that clinical CD represents only the tip of the iceberg. The subclinical disease is not infrequent in the general population, and serological tests such as tTGA can be used as markers for the identification of these asymptomatic individuals.[8] In several studies, the sensitivity and specificity of this test compared with biopsy-proven disease were 94% and 98%, respectively.[9–11] This is important because the treatment of asymptomatic patients with T1DM having a gluten-free diet seems to have a positive effect on glycemic control and on the growth. Furthermore, it can prevent osteoporosis and the development of autoimmune diseases.[12] The aim of our study was to determine the prevalence of CD in patients with T1DM using tTGA as a screening test.

## Methodology

For the current study, 100 patients with T1DM (58 women and 42 men) from diabetes clinic of Medical University of Tabriz, Iran, as study population and 150 nondiabetic healthy people (82 women and 68 men) without having autoimmune and other CD-related diseases as control population were recruited. All patients were interviewed by the doctor about any history of diseases and symptoms compatible with CD and a questionnaire was filled out. After formal consenting, 7 ml of blood was collected from each subject. Samples were centrifuged and the serum was separated, divided into two aliquots and immediately stored at −20°C. Anti-tissue transglutaminase IgA antibodies were determined by enzyme-linked immunosorbent assay (ELISA) with human recombinant tTGA as antigen, using a commercial kit (Eu- tTG IgA, Eurospital, Trieste, Italy). Results were considered positive when the tTGA levels were higher than 7 AU/mL. The tTGA serological test is not appropriate for patients with IgA deficiency and due to the prevalence of 2–3% IgA deficiency in general population,[13] the serum IgA levels should be determined before any serological tests such as tTGA. This helps in eliminating false-negative results. The total serum IgA levels was determined by turbidimetry and IgA deficiency was considered positive when the IgA levels were <70 ng/dl. The tTGA positive and, IgA deficient subjects were clinically evaluated and submitted to upper gastrointestinal endoscopy. Crypt hyperplasia and villous atrophy (VA) were classified as either partial (PVA) or total (TVA), according to Marsh.[14]

The Statistical Package for Social Science (SPSS), version 11.5, was used for the statistical analysis. Simple statistics such as frequency, mean and standard deviation were used. Also, chi-square, t-test and Mann-whitney U test were used for comparison. The results were considered to have a statistical significance when the *P* values were <0.05.

## Results

Serological screening for CD based on tTGA was performed in 100 patients with T1DM (58 women and 42 men) aged 21.8 ± 8.86 years (age range: 7–50 years), and in 150 healthy controls (82 women and 68 men) aged 28.9 ± 9.07 years (age range: 4–50 years).

**Figure 1:**
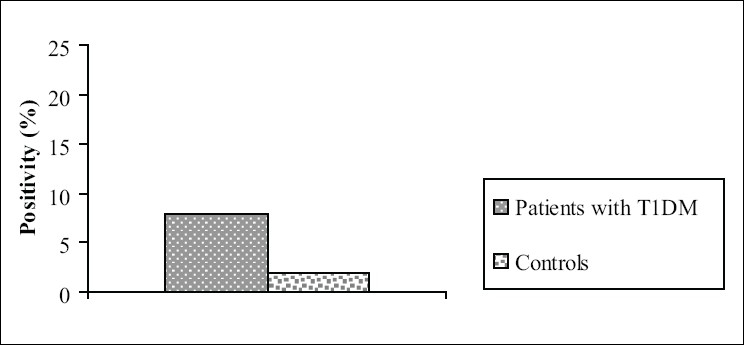
Tissue trnsglutaminase antibodies (tTGA) in patients with type 1 diabetes and controls

The results for patients with T1DM and controls are shown in Figure 1 Statistically significant positivity of tTGA was observed in the T1DM patients when compared to the controls (*P* = 0.023). Eight patients, three men and five women, were positive for tTGA, while three of the 150 control individuals (2%), one man and two women, were positive too.

Table 1 shows demographic and clinical characteristics of subjects with DM compared with healthy controls. There was no difference based on gender between cases and controls. However, the mean age of controls was significantly higher than the patients with T1DM (*P* < 0.001). Type 1 patients with DM reported positive history of chronic diarrhea and autoimmune disease significantly more often than controls (*P* = 0.006 and *P* = 0.001, respectively). Four percent of T1D patients (n = 4) and 1.3% of controls were IgA deficient (*P* > 0.05), but none of the tTGA positive individuals in both the groups had a IgA titer of <70 ng/dl.

**Table 1:** Demographic and clinical characteristics of patients with type 1 diabetes compared with healthy controls

Characteristics	Patients*	Healthy controls**	*P* value
Sex (female/male)	58/42	82/68	0.603
Age (mean ± SD)	21.85 ± 8.86	28.91 ± 9.07	0.001
FBS mg/dl (mean ± SD)	184/86 ± 75/03	77.46 ± 10.78	< 0.001
IgA deficiency (Pos/Neg)	4/96	2/148	0.177
History of chronic diarrhea (Y/N)	5/95	0/150	0.006
History of anemia (Y/N)	10/90	10/140	0.341
History of autoimmune disease (Y/N)	7/93	0/150	0.001

* n = 100

** n = 150, FBS: fasting blood sugar; SD: standard deviation; Pos: positive; Neg: negative; Y: yes; N: no

Table 2 represents characteristics of tTGA positives compared with tTGA negatives in patients with T1DM. The mean age of tTGA positive cases was significantly lower than tTGA negative ones (mean difference 7.17; 95% CI: 0.82–13.52 years). Furthermore, the mean age of diabetes diagnosis was lower in tTGA positive than tTGA negative subjects, but was not statistically significant (*P* > 0.1). Positive history of dermatitis was reported in only one of eight tTGA positive cases (*P* = 0.001). Of the tTGA and IgA deficient patients with T1DM only three (all of them were tTGA positive) underwent duodenal mucosa biopsy. The biopsy showed PVA or TVA in all three patients.

**Table 2:** Characteristics of tTGA positives compared with tTGA negatives in T1DM patients

Characteristics	tTGA positive T1DM patients*	tTGA negative T1DM patients**	*P* value
Sex (female/male)	5/3	53/39	0.788
Age (mean ± SD)	15.25 ± 5.54	22.42 ± 8.88	0.027
FBS mg/dl (mean ± SD)	172.40 ± 36.81	185.79 ± 77.21	0.703
IgA deficiency (Pos/Neg)	0/8	4/88	0.547
History of chronic diarrhea (Y/N)	0/8	5/87	0.499
History of anemia (Y/N)	0/8	10/82	0.326
History of ketoacidosis (Y/N)	1/7	29/63	0.260
History of dermatitis (Y/N)	1/7	0/92	0.001
History of autoimmune disease (Y/N)	1/7	6/86	0.525

* n = 8

** n = 96; FBS: fasting blood sugar; SD: standard deviation; Pos: positive; Neg: negative; Y: yes; N: no

## Discussion

The prevalence of CD in patients with T1DM who underwent tTGA testing was 8%. There was a significant difference in frequency of the tTGA positivity between cases and controls. Results of studies in Western, African and Middle-East countries showed high variation of CD prevalence in patients. In European and American countries, the prevalence ranged from 1–8% by serology.[15] A recent study conducted in UK, of total 113 children and adolescents with T1DM, 6.2% were tested antibody positive.[16] In addition, 12.3% of Danish children with T1DM were positive for CD.[17] These values were remarkably higher among Africans. The prevalence of CD in Libya and Algeria was 21.3% and 16.3% respectively.[18,19] In the Middle-East countries, positive serology tests for CD was detected in 20.9% of Saudi children with T1DM.[20] Apparently, the prevalence of the disease in the present study (8%) is similar to those reported in European countries using serological tests. However, this prevalence is relatively higher than those previously reported in Iran among patients with T1DM. In a study by Shahbazkhani *et al*,[21] EMA was positive in 2.4% of the patients. Two other studies in Iran compared CD prevalence between the cases (with T1DM) and controls (without T1DM). In one of them, 3.8% of 80 patients with T1DM had positive serology test for CD[22] and in other study, 3.3% of patients with T1DM were tTGA positive,[23] but unlike in our study, these data were not significantly different between cases and controls. The higher CD prevalence in the present study might be explained by differences in study conditions: (1) present study was performed in northwest of Iran; therefore, genetic and environmental factors might account for some of the regional differences, (2) we used tTGA which is proven to be a very specific indicator for CD in contrast with other studies wherein either AGA or EMA was used. Consequently, the prevalence of CD determined by tTGA would be higher than those determined by either AGA or EMA as a screening test. The test for IgA antibodies against tTGA is proven to be highly accurate, the ELISA that has less potential for interpretational error and thus, represents an improvement over the EMA that relies on indirect immunofluorescence, thus, carrying an inherent intraobserver subjectivity in interpretation of the test.[11] It seems that the lower prevalence of CD in patients with T1DM found in previous studies in Iran might be underestimates of the true prevalence in the population. Also, the observed prevalence of CD in our study might have been underestimated. Had all tTGA positive patients with T1DM and IgA deficient ones underwent duodenal biopsy, the true prevalence of CD might have been >8%. Although not all tTGA positives in our study underwent an intestinal biopsy, the confirmed CD prevalence in patients with T1DM was still high (3%) compared with the general population (0.86%).[24]

Since the control group was representative of general population, where CD may develop at any age, both during childhood or adolescence and is relatively common in the adult and elderly patients,[3,25–27] the significant higher mean age of controls than cases does not alter the significant difference of CD prevalence between them.

In the present study, the age of diabetes mellitus diagnosis was lower in tTGA positive subjects compared to tTGA negative ones in patients with T1DM, but the difference was not statistically significant. However, the tTGA positive cases had a significant lower age than tTGA negative cases. These observations are in agreement with some studies which revealed that the risk of CD and T1DM is higher in younger age groups than in older ones.[17,28,29]

Dermatitis is reported to occur in CD patients especially between 15 and 40 years.[7] In our research, one out of eight (12.5%) tTGA positive patients with T1DM had a positive history of dermatitis (*P* = 0.001). The autoimmune disease history did not differ between the tTGA positives and tTGA negatives in with T1DM patients and the significant difference in having history of autoimmune diseases between cases and controls might be described by the fact that the risk of autoimmune disease increases in patients with T1DM.[30]

Diarrhea is another common symptom among CD patients, but none of tTGA positive patients in our study reported chronic diarrhea. As mentioned before, tTGA screens patients with latent CD, in whom there is absence of diarrhea.[31] Furthermore, Iranian diet contains wheat as a major component, therefore, exposure to a high level of wheat proteins induces some degree of immune tolerance, leading to milder symptoms. This observation supports recommendation of CD screening in patients with T1DM. It should be noted that the significant difference of chronic diarrhea history between the patients with T1DM and the controls was not the result of age difference between the two groups because there was no association between age and reporting history of chronic diarrhea in our research. Probably this finding is due to higher prevalence of chronic diarrhea in patients with T1DM. In a study performed by Lysy *et al*, nondiabetic and diabetic diarrheas have high prevalence in T1D patients and the most common cause of nondiabetic diarrhea is drug therapy.[32]

Longitudinal prospective studies compared with cross-sectional ones can better show the true prevalence of CD in patients with T1DM. Moreover, the benefits of a gluten-free diet (GFD) in these patients are not well established in Iran. So, it is necessary to conduct a short-term and long-term clinical randomized control trials to investigate the effect of GFD. Obviously, since most of the patients with CD are asymptomatic, many studies recommend serologic testing for diagnosis of T1D and every two years after that.[30,33,34] Thus, the younger individuals with T1DM could benefit from GFD to improve their quality of life.

## Conclusion

This study showed the higher prevalence of CD in patients with T1DM than the general population in northwest of Iran and the data lend support to recommend regular screening for CD in all patients with T1DM.
